# Gigantic and Continuous Output Power in Ionic Thermo‐Electrochemical Cells by Using Electrodes with Redox Couples

**DOI:** 10.1002/advs.202303407

**Published:** 2023-08-01

**Authors:** Wencong Zhang, Liyu Qiu, Yongjian Lian, Yongqiang Dai, Shi Yin, Chen Wu, Qianming Wang, Wei Zeng, Xiaoming Tao

**Affiliations:** ^1^ Key Laboratory of Theoretical Chemistry of Environment Ministry of Education School of Chemistry South China Normal University Guangzhou 510006 China; ^2^ The center of flexible sensing technology Institute of Chemical Engineering Guangdong Academy of Sciences Guangzhou 510665 China; ^3^ MOE & Guangdong Province Key Laboratory of Laser Life Science & Institute of Laser Life Science Guangzhou Key Laboratory of Spectral Analysis and Functional Probes College of Biophotonics South China Normal University Guangzhou 510631 China; ^4^ Research Institute for Intelligent Wearable Systems The Hong Kong Polytechnic University Hong Kong China

**Keywords:** carnot‐relative efficiency, ionic thermo‐electrochemical cells, output energy density, redox reactions on electrode, thermodiffusion effect in hydrogel

## Abstract

The main obstacle of ionic thermo‐electrochemical cells (TECs) in continuous power supply lies in a low heat‐to‐electricity energy conversion efficiency because most TECs work in thermodiffusion mode in which the ions are confined in a liquid/electrolyte media. The introduction of the redox couple onto the electrode surface may overcome the obstacle by resolving the low mass transport rate of ions caused by the redox process occurring near but not on the electrode surface. Herein, the authors demonstrate enhancement of TECs by integrating the redox couple directly onto the electrode surface to maximize the mass transport efficiency. A discontinuous interfacial modification strategy is developed by using a carbon cloth/iron (II/III) phytate as the symmetric electrodes. The gelled electrolyte consisting of a polyacrylamide matrix and phytic acid is shown to promote selective ion diffusion. A synergistic combination consisting of the thermodiffusion effect and redox reactions on the electrode is established in a pre‐treated layout. Such TEC affords a high output voltage of 0.4 V, an excellent instantaneous output power density (20.26 mW m^‐2^ K^‐2^) and a record‐high 2 h output energy density (2451 J m^‐2^) under T_H_ = 30 °C with T_C_ = 15 °C, with an ultrahigh Carnot‐relative efficiency of 1.12%.

## Graphical abstract

The continuous output power and Carnot‐relative efficiency of ionic thermo‐electrochemical cells (TECs) can be improved by the utilization of electrodes with redox couples. The introduction of symmetrical electrodes or asymmetric electrodes can demonstrate the universality of electrodes with redox couples for TECs. The application of Grotthuss principle can further improve the performance of TECs.

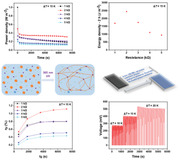

## 1 Introduction

Low‐grade heat (<100 °C), one of the most common forms of energy in nature, is a potential source of energy^[^ ^1^, ^2^ ^]^ whose efficient utilization could contribute to the goals of carbon dioxide peaking and carbon neutrality. A large amount of low‐grade heat is present in industrial production, the environment, solar thermal energy devices, etc.^[^ ^3^, ^4^, ^5^, ^6^, ^7^, ^8^, ^9^, ^10^, ^11^, ^12^, ^13^ ^]^ Solid‐state ionic thermo‐electrochemical cells (TECs) have shown great promise in converting low‐grade heat into electricity with the advantages of high thermopower,^[^ ^11^, ^14^, ^15^ ^]^ low cost,^[^ ^4^, ^16^, ^17^ ^]^ and module flexibility.^[^ ^18^, ^19^, ^20^, ^21^, ^22^ ^]^ At present, TECs working in capacitor's fashion or thermodiffussion mode only offer low continuous electric energy output for a limited time.^[^ ^7^, ^15^, ^23^ ^]^ One of the reasons for this is that ions cannot pass through the electrodes in the external circuit as well as the accumulated electrode surface.^[^ ^14^, ^24^ ^]^

Combining ionic thermodiffusion with the thermogalvanic effect has been demonstrated to be an efficient way to boost power output and its continuity.^[^ ^14^, ^24^, ^25^ ^]^ Originating from the entropy change of the redox reactions of K_3_Fe(CN)_6_/K_4_Fe(CN)_6_ under a temperature gradient, the thermogalvanic effect works synergistically with the p‐type thermodiffusion effect and greatly improves thermoelectric performances.^[^ ^14^, ^24^, ^25^ ^]^ Although the synergistic effect of thermodiffusion and thermogalvanic appears to dominate the thermoelectric conversion process, the resultant entropy change could be still influenced by a variety of factors, including charge transfer overpotentials caused by the contact of the redox couple at the electrode interface and mass transport overpotentials relevant to the migration or structural change of redox species in electrolytes.^[^ ^4^ ^]^ The crucial issue of using TECs for continuous power supply is that the thermally diffused ions are confined in the liquid/electrolyte medium and separated from external circuitry,^[^ ^14^, ^24^ ^]^ resulting in poor thermoelectric conversion efficiency. Therefore, integrating the redox couple on the electrode surface effectively enhances the mass transport rate of ions and the redox process on the electrode surface, potentially improving the thermoelectric conversion efficiency of TECs. Recent work demonstrating that output power performance can be enhanced by attaching polymer redox polyaniline (PANI) to a carbon weaved fabric (CWF) electrode supports this idea.^[^ ^26^ ^]^ In addition, the introduction of carbon nanotubes,^[^ ^27^ ^]^ carbon nanotube aerogels,^[^ ^28^ ^]^ porous electrodes,^[^ ^29^ ^]^ anisotropic holey graphene aerogel electrodes,^[^ ^30^ ^]^ a graphite paper electrode,^[^ ^31^ ^]^ and the integration of thermoelectric conversion with reverse electrodialysis^[^ ^32^ ^]^ into thermocell can increase the instantaneous power density (*P*_max_/Δ*T* ^2^).^[^ ^24^, ^33^ ^]^ However, little effort has been made on the continuous output energy performances of the thermocell.^[^ ^34^ ^]^ In 2019, Hu et al. suggested a different mode of directly connecting the thermocell with load resistance after the voltage build‐up stage.^[^ ^35^ ^]^ The method has been used in some studies.^[^ ^11^, ^36^, ^37^, ^38^, ^39^ ^]^ Finally, it must be mentioned that assembly strategy can also affect the performance of the thermocell.^[^ ^40^ ^]^

Herein, we employed a discontinuous interface modification strategy to load redox‐pairs onto symmetric and asymmetric electrodes. We have experimentally demonstrated an exceptional example of boosting the instantaneous and continuous output performance of the TEC by attaching a redox‐active couple (iron (II/III) phytate) onto carbon cloth electrodes. The TEC worked with the thermodiffusion effects of both the ionic hydrogel (middle layer) and the redox reactions of the carbon cloth/iron (II/III) phytate electrode (top and bottom layer). The experimental results showed that the TEC with symmetrical electrodes has an excellent instantaneous output power density (20.26 mW m^−2^ K^−2^), a record‐high 2 h output energy density (2451 J m^−2^) when *T*_H_ = 30 °C and *T*_C_ = 15 °C, and an ultrahigh Carnot‐relative efficiency of 1.12%.

It has been well documented that the instantaneous and continuous output performance depends heavily on the dispersion of the redox couple.^[^ ^4^ ^]^ The output power density would be largely suppressed at a low ionic concentration. The most significant change employed in this study involved transferring the redox pair onto solid electrodes so that the influencing factors related to entropy changes, such as the structural instability of redox species, mass transport overpotentials and interaction effects from the solvents, could be avoided. Regarding the charge transport, the possible energy filtering effect in the dispersion phase and host interface could be considered. The assembled composite electrode acted as both the reactive electrode surface and the high current collector.

Furthermore, to verify the reliability and intrinsic mechanism of entropy change induced by electron transfer during the electrochemical reaction, ferrocene has been chosen as the alternative for redox pair since the ferrocene/ferrocenium couple is the most suitable standard in organometallic electrochemistry. After the integration of the above symmetric (iron (II/III) phytate) and asymmetric electrodes (ferrocene/ferrocenium), a variety of questions in TECs‐design, including energy transfer efficiency, power density, and the stability issue have been addressed. As proof of concept, the practical working performance has been demonstrated in a module consisting of six thermo‐electrochemical cells with symmetric electrodes. This device could generate a voltage of 2.3 V at a temperature difference of 15 °C (*T*_H_ = 30 °C, *T*_C_ = 15 °C) and efficiently power the high‐brightness of LEDs. This study elucidates the electrode‐electrolyte interface architecture and contributes to its transformation from lab‐scale module to market‐relevant device.

## 2 Results and Discussion

### 2.1 Design Principle and Characterization of TECs with Symmetrical Electrodes

**Figure 1:**
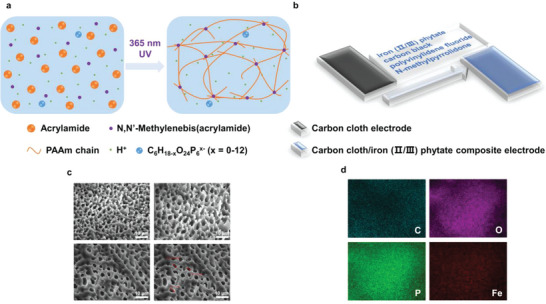
Design principle and characterization of TECs with symmetrical electrodes. a) Schematic diagram of the structure of the ionic hydrogel. b) Schematic diagram of the composition of the carbon cloth/iron (II/III) phytate composite electrode. c) Internal microstructure of the ionic hydrogel. d) Elemental mappings of the carbon cloth/iron (II/III) phytate composite electrode.

A hierarchical structure possessing three functional layers (referring to as top, middle and bottom) has been designed for TECs with symmetrical electrodes. The polyacrylamide ionic hydrogel serves as the middle layer in the sandwich‐type structure, generating voltage via the thermodiffusion effect of the charge carrier based on phytic acid under a thermal gradient (**Figure** **1**a). As for the assembly of the top and bottom layers (Figure 1b), the formation of carbon cloth/iron (II/III) phytate composite electrodes fully utilizes redox reactions through the entropy change of the redox couple with a concentrated solid sample, and optimized output current density with fast electron transfer can be expected.

A simple and effective layer‐by‐layer strategy has been employed, and the specific preparation details can be found in the experimental section. The electrical and charge migration properties are greatly dependent on shape and size, so the performance of thermoelectric materials can be optimized by control over the microstructure. One typical characteristic for the interior nature of the ionic hydrogel is its large pore structure with an average diameter of 3–4 µm (Figure 1c). Making use of this arrangement, the ion diffusion rate has been reinforced. The power limiting factors will be minimized. The facile mild‐solution‐based method at room temperature sheds new light on the control synthesis of electrode layers in nanoscale domains and the composite interface consists of iron (II/III) phytate, carbon black, and carbon cloth (Figure S1, Supporting Information). The SEM image (Figure S2, Supporting Information) region of the carbon cloth/iron (II/III) phytate composite electrode was employed for element mapping in Figure 1d. The presence of C, O, P, and Fe with their distributions has been verified in the elemental mapping images (Figure 1d). Accordingly, the combinative strategy based on composite electrode, electrolyte additive (phytic acid), and quasi‐solid‐state electrolyte allows for the efficient electron transfer kinetics of the redox couple onto solid support and increases the range of reaction sites due to high surface area.

### 2.2 Mechanistic Analysis of TECs with Symmetrical Electrodes

The fast ion transport in electrolyte media is controlled by the thermodiffusion effect, and the ionic charge carriers can behave differently under the stimulation of the thermal gradient. Essentially, the proton derived from phytic acid has no outside electron shell and shows strong affinity to the electron density of its neighboring structure. Two representative cases explain the proton transportation process. The first mechanism relies on proton migration assistance from the translational dynamics of the hosting species as the vehicle. Another reorganization of the proton environment is termed the Grotthuss principle,^[^ ^41^, ^42^, ^43^ ^]^ in which the protons diffuse through hydrogen bonds from one vehicle to the adjacent one. We hypothesized the latter case would be more inclined to support proton diffusivity since the Grotthuss‐type vehicles gave rise to pronounced local dynamics but were not allowed to leave their own sites. The predominant gel‐electrolyte source‐polyacrylamide possesses numerous hydrogen bonding acceptors and donors in its long polymeric chain. The protons could be involved in the interaction with the adjacent oxygen atoms from amide groups, and the asymmetrical hydrogen bond (O─H─O) with directional nature would be formed. Such synergistic effect from polyelectrolyte might favor the diffusion rate, rotation, and reorientation of protons. In this case, the proton hopping assisted by the interaction with amide units in the soft chain causes the fast proton conductivity and leads to a sharp difference between the thermodiffusion rate of positive (H^+^) and negative (phytate) charges. During this process, the performed density functional theory (DFT) calculation should be considered since the results reveal the extension and growth of repetitive polyacrylamide units possess a stronger binding energy for H^+^ to ‐CONH_2_ (Table S1, Supporting Information). In this way, the acrylamide trimer, compared to the acrylamide monomer unit, shows higher affinity to the proton. Therefore, the migration path entangled along with the polyacrylamide chain is reliable. Grotthuss proton conduction is very fast, and it is responsible for the high thermopower of TECs. It is worth noting that the presence of hydrogen as a constituent of water enables fast conduction of protons in aqueous systems. The transportation of proton from one molecule to its neighboring one has been realized by the operation of rearrangements along with the water chain.^[^ ^7^ ^]^ Evidently, there is no moving or translational shuttle units during the assistance process of proton migration, and proton transport with a vehicle mode should not be included. In current studies, the host polymeric segment demonstrates its local dynamics and resides on confined sites. Based on the above discussion, we designate the main proton diffusion mode as a Grotthuss type.

**Figure 2:**
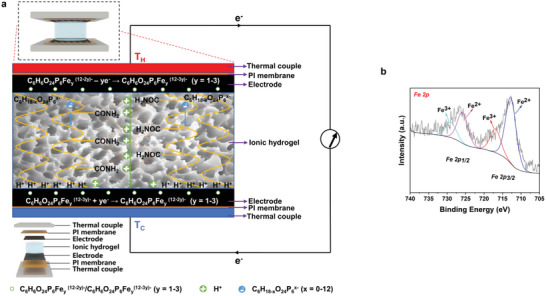
Mechanistic analysis of TECs with symmetrical electrodes. a) Schematic diagram of TECs with symmetrical electrodes. b) XPS Fe 2p spectrum of the carbon cloth/iron (II/III) phytate composite electrode.

Therefore, the general mechanistic analysis of TECs with symmetrical electrodes can be described as follows（Figure 2a）: both H^+^ and C_6_H_18−_ *_x_* O_24_P_6_ *^x^* ^−^ are dispersed in ionic hydrogel, but the fast migration rate of H^+^ and the cooperativity of the hydrogen bonding interaction drive the efficient movement of cations toward the cold side, confining many anions (C_6_H_18−_ *_x_* O_24_P_6_ *^x^* ^−^) to the hot side. The accumulation of protons at the cold side attracts the transmission of electrons near the interface of the carbon cloth/iron (II/III) phytate composite electrodes, which is beneficial to the reduction reaction of C_6_H_6_O_24_P_6_Fe*_y_* ^(12−3^ *^y^* ^)−^. In an analogous manner, the highly concentrated C_6_H_18−_ *_x_* O_24_P_6_ *^x^* ^−^ at the hot side induces vacancies at the anode, which leads to the oxidation reaction of C_6_H_6_O_24_P_6_Fe*_y_* ^(12−2^ *^y^* ^)−^. The specific redox reactions are given here:

(1) $$\begin{array}{ccc} & & \mathrm{At}\;\;\mathrm{the}\;\;\mathrm{hot}\;\;\mathrm{side}:\\ & & C_{6}H_{6}O_{24}P_{6}Fe_{y}^{\left(12-2y\right)-}-\;ye^{-}\to \;C_{6}H_{6}O_{24}P_{6}Fe_{y}{{}^{\left(12-3y\right)}}^{-}\left(y\;=\;1-3\right) \end{array}$$

(2) $$\begin{array}{ccc} & & \mathrm{At}\mathrm{the}\mathrm{cold}\mathrm{side}:\\ & & C_{6}H_{6}O_{24}P_{6}{\mathrm{Fe}}_{y}{{}^{\left(12-3y\right)}}^{-}+\;ye^{-}\to \;C_{6}H_{6}O_{24}P_{6}{\mathrm{Fe}}_{y}{{}^{\left(12-2y\right)}}^{-}\left(y\;=1-3\right) \end{array}$$

The Fe^2+^ in C_6_H_6_O_24_P_6_Fe*_y_* ^(12−2^ *^y^* ^)−^ loses an electron and is transformed to Fe^3+^ in C_6_H_6_O_24_P_6_Fe*_y_* ^(12−3^ *^y^* ^)−^ with the stable half‐full d^5^ electrons, while the Fe^3+^ in C_6_H_6_O_24_P_6_Fe*_y_* ^(12−3^ *^y^* ^)−^ with its high charge and small radius can easily retrieve an electron to be reduced to Fe^2+^ in C_6_H_6_O_24_P_6_Fe*_y_* ^(12−2^ *^y^* ^)−^, thus forming a naturally associated redox pair. The structural information of the carbon cloth/iron (II/III) phytate composite electrode has also been evaluated by X‐ray photoelectron spectroscopy (XPS) (Figure S3, Supporting Information). Except the elemental binding energies of C 1s, O 1s, and P 2p, the presence of Fe^3+^ and Fe^2+^ can be observed in the active layer, which has provided evidence to verify the existence of the redox couple (**Figure** **2**b). Due to the deposition of the electro‐active species onto solid support, high power performance is realized by fast ion movement to the electrode surface.

### 2.3 Thermoelectric Conversion Performance of TECs with Symmetrical Electrodes

**Figure 3:**
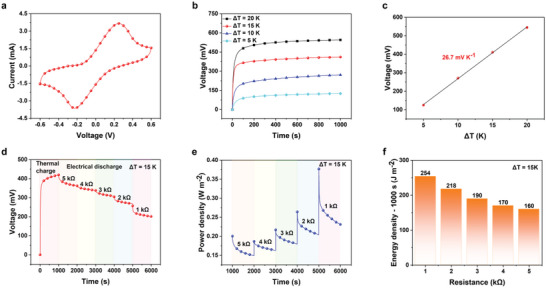
Thermoelectric conversion performance of TECs with symmetrical electrodes. a) CV curves of the TECs. b) Open‐circuit voltage of the TECs under variable *T*_H_ (20, 25, 30, and 35 °C) with a fixed *T*_C_ = 15 °C. c) Thermopower of the TECs. d) Thermal charge and electrical discharge of the TECs under *T*_H_ = 30 °C and *T*_C_ = 15 °C. e) Output power density of the TECs at different external resistances under *T*_H_ = 30 °C and *T*_C_ = 15 °C for 1000 s. f) Corresponding energy density of the TECs at different external resistances under *T*_H_ = 30 °C and *T*_C_ = 15 °C for 1000 s.

The redox process related to the generation of electricity should be performed in a reversible manner for sustainable operation. The electrochemical behavior has been explored in TECs with symmetrical electrodes. In cyclic voltammetry analysis, oxidation and reduction peaks with high symmetry are observed, and the well‐reversible redox reaction is achieved (**Figure** **3**a). Figure S4, Supporting Information shows that the shift in potential at different temperatures is insignificant, and the reaction entropy is not large. In that case, the high thermopower is mainly due to the ionic Seebeck coefficient (ionic thermodiffusion effect). In addition, ionic conductivity (*σ*_i_) was evaluated using electrochemical impedance spectroscopy (EIS). In Figure S5, Supporting Information, all the Nyquist plots display straight lines without semicircles in the high‐frequency region. This could be caused by the dielectric relaxation of ions offsetting the capacitive response due to high ion loading. The ionic resistance, which is the intercept of the line on the horizontal axis according to the equivalent circuit of the Nyquist diagram, has been recorded to obtain the ionic conductivity of TECs. The determined ionic conductivity possesses high electrical conductivity, and the *σ*_i_ value are 0.75 S m^−1^ (TECs with carbon cloth electrodes), 1.72 S m^−1^ (TECs with asymmetric electrodes) and 2.78 S m^−1^ (TECs with symmetrical electrodes), as shown in Figure S6, Supporting Information. Electrons are trapped and lost on the solid electrode regardless of the gel resistance, the viscosity of electrolyte and the presence of additives, thus being conducive to thermocell conversion efficiency. Moreover, the functions of carbon black and carbon cloth promoting electron transfer due to their high specific surface area cannot be ruled out. When the model system is exposed to temperature change, the thermodiffusion of H^+^ leads to a net positive charge near the cold side, generating an electric field directing from the cold to the hot side. To verify the diffusion of phytic acid, the parallel experiments to compare H^+^ concentration at cold/hot ends of the hydrogels were carried out after thermovoltage reached the stabilized value. In Figure S7, Supporting Information, the cold half of the hydrogel exhibited more consumption of a NaOH solution, whereas the effect of the hydrogel at a relatively higher temperature was much less intensive. These results indicate that C_6_H_18−_ *_x_* O_24_P_6_ *^x^* ^−^ was diffused in the gel system and mainly localized in the hot side. Intrinsically, such electrolytes in the absence of a redox species (only phytic acid in ionic hydrogel) have difficulty in providing energy continuously. Nevertheless, the vacancies and electrons on the carbon cloth/iron (II/III) phytate composite electrodes strengthen the redox reactions of iron (II/III) phytate so that the TECs with symmetrical electrodes generate an open‐circuit voltage. More specifically, when Δ*T* = 5, 10, 15, and 20 K (variable *T*_H_ = 20, 25, 30, and 35 °C versus a fixed *T*_C_ = 15 °C), the measured open‐circuit voltage has been recorded as 125, 271, 411, and 545 mV, respectively (Figure 3b). In addition, the TECs adequately work at nearly 60 °C (Figure S8, Supporting Information). To note, voltage versus time was 10 points per second after the temperature gradient was applied to the electrode. After the voltage stabilized, the voltage build‐up process was complete. A short circuit was then applied to the sample again, and a fast recovery was recorded. The data of voltage versus temperature gradient was recorded and used to calculate the thermopower of TECs. In this manner, their thermopower was determined as 26.7 mV K^−1^ within a range of 15 to 35 °C (Figure 3c).

The heat‐to‐electricity conversion process was explored by thermal charging the TECs with symmetrical electrodes under *T*_H_ = 30 °C and *T*_C_ = 15 °C to reach a near‐saturated voltage of 411 mV. The voltage profile upon electrical discharging for 1000 s was recorded at the same Δ*T* with an external resistance of 5, 4, 3, 2, and 1 kΩ (Figure 3d). The output voltage demonstrates its dependence on reduction in the external resistance, achieving a constant value in a stable operation mode. Without the assistance of the redox pair on the electrode, the large potential difference achieved at the initial stage dissipated due to the connection of an external circuit, and the performance of TECs with carbon cloth was significantly inferior to that of TECs functionalized by symmetrical electrodes (Figures S9 and S10, Supporting Information). Additionally, both the power density and energy density exhibit negative correlations with the external resistance values in a time duration of 1000 s (Figure 3e,f).

### 2.4 Instantaneous and Continuous Output Performance of TECs with Symmetrical Electrodes

**Figure 4:**
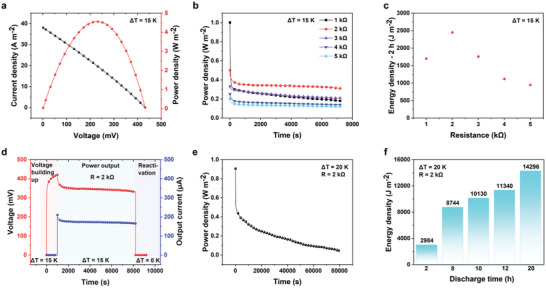
Instantaneous and continuous output performance of TECs with symmetrical electrodes. a) Voltage and output power density versus output current density of the TECs under *T*_H_ = 30 °C, *T*_C_ = 15 °C, and a sweep speed of 100 mV s^−1^. b) Output power density of the TECs for 2 h at different external resistances under *T*_H_ = 30 °C and *T*_C_ = 15 °C. c) Corresponding energy density of the TECs for 2 h at different external resistances under *T*_H_ = 30 °C and *T*_C_ = 15 °C. d) The measured voltage and current curves of the TECs at external resistance (2 kΩ) under *T*_H_ = 30 °C and *T*_C_ = 15 °C. e) Output power density of the TECs under *T*_H_ = 35 °C and *T*_C_ = 15 °C. f) Energy density of the TECs under *T*_H_ = 35 °C and *T*_C_ = 15 °C.

After the evaluation of the redox species and ionic hydrogel type electrolyte, the TECs with symmetrical electrodes achieved an ultra‐high instantaneous output power density (20.26 mW m^−2^ K^−2^) at a 100 mV s^−1^ sweep speed (**Figure** **4**a), which is 11.6 times that of the controlled sample (TECs with carbon cloth) (Figure S11, Supporting Information). The instantaneous output power density at different sweep speeds is shown in Figure S12, Supporting Information, and the instantaneous output power density remains at a high value even after the sweep speed is lowered. To some extent, IV scans can reflect the instantaneous output performance of the TECs but cannot reflect its continuous output performance.^[^ ^44^, ^45^ ^]^ Thus, we adopted a more reasonable method to evaluate the continuous output performance of the TECs that involved connecting the TECs with a sequence of constant resistances (or resistive loads). As illustrated in Figure 4b, the continuous output was tested by connecting the thermocell with the external resistances from 5 to 1 kΩ for 2 h under *T*_H_ = 30 °C and *T*_C_ = 15 °C. The measured energy density loaded by the external resistances gave rise to parabolic behavior (Figure 4c) and reached a maximum value of 2451 J m^−2^ in the presence of 2 kΩ resistance, which increased more than 20‐fold and far surpassed the value of the TECs with carbon cloth (Figure S13, Supporting Information). During the temperature elevation process, the thermo‐ionic charging treatment drives the positive/negative ionic charges to gather near each electrode to generate the electric field. As provided in Figure 4d, the voltage was raised to 422 mV in 1000 s under Δ*T* = 15 K. When the model system connects with an external load in circuit, the movable electrons can flow in the closed circle and balance the voltage generated by the separated ions. Accordingly, the continuous power output was realized by appending the TECs with symmetrical electrodes under an external resistance of 2 kΩ. Similarly, continuous power output was also demonstrated by assembling the TECs with symmetrical electrodes in the presence of variant external resistances (1, 3, 4, and 5 kΩ), as shown in the Figures S14–S17, Supporting Information. The electronic discharging process can be extended to 2 h with high stability and no significant degradation for voltage observed. In the last stage, the cations and anions accumulated onto the two electrodes would gradually return to their original positions due to the disconnection of the external circuit and the removal of the temperature gradient. Here, the voltage declined to zero in a short‐circuit status. Concerns about the long‐term stability were also allayed when the thermocell was subjected to discharge with a constant external resistance of 2 kΩ for 80 000 s (Δ*T* = 20 K) (Figure 4e). The energy density significantly increased due to the extension of the continuous output time, reaching as high as 14 296 J m^−2^ for 20 h (Figure 4f). Such experimental results demonstrate that continuous power output necessitates maintaining highly concentrated reactants adjacent to anode/cathode and that the electrode should be restructured to accelerate the migration process and downsize the mass transport resistance. Therefore, this discontinuous deposition approach of the electrode with the redox couple as a functional coating displays a stable power output in device and prevents the traditional detrimental effects associated with diffusion‐path problems occurring at electrode‐electrolyte interfaces.

### 2.5 Instantaneous and Continuous Output Performance of TECs with Asymmetric Electrodes

**Figure 5:**
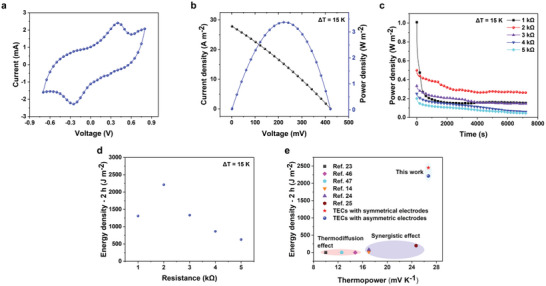
Instantaneous and continuous output performance of TECs with asymmetric electrodes. a) CV curves of the TECs. b) Voltage and output power density versus output current density of the TECs under *T*_H_ = 30 °C and *T*_C_ = 15 °C. c) Output power density of the TECs for 2 h at different external resistances under *T*_H_ = 30 °C and *T*_C_ = 15 °C. d) Corresponding energy density of the TECs for 2 h at different external resistances under *T*_H_ = 30 °C and *T*_C_ = 15 °C. e) Performance comparison between this work and previously reported TECs based on the thermodiffusion effect and the synergistic effect of thermodiffusion and thermogalvanic cells.

Ferrocene has caused considerable interest for its stability and unparalleled electron transfer kinetics, Herein, the ferrocene/ferrocenium couple onto carbon cloth was recorded as the asymmetric electrode since this redox pair has always been considered to be internal standard in organometallic electrochemistry. In **Figure** **5**a, its capability to undergo reversible one‐electron oxidation/reduction was demonstrated. The peak at 0.40 V was assigned to the oxidation reaction as: [Cp_2_Fe] – e^−^ → [Cp_2_Fe]^+^, while the signal at −0.25 V could be corresponding to the reduction reaction as: C_6_H_6_O_24_P_6_Fe*_y_* ^(12−3^ *^y^* ^)−^ + ye^−^ → C_6_H_6_O_24_P_6_Fe*_y_* ^(12−2^ *^y^* ^)−^ (*y* = 1–3). Its instantaneous power density (15.02 mW m^−2^ K^−2^) was determined to be 860% higher than that of the TECs with carbon cloth (Figure 5b and Figure S11, Supporting Information) and was comparable to that of the system integrated with the symmetric electrode (Figure S18, Supporting Information). According to measurement of continuous output performance with external resistances 5 to 1 kΩ for 2 h under Δ*T* = 15 K, the accumulated energy density reaches its maximum value (2213 J m^−2^) at an external resistance of 2 kΩ (Figure 5c,d). Its substantial increasing effect in contrast to the blank sample (24.3 times that of TECs with carbon cloth) was observed (Figure S13, Supporting Information), and the energy density output is slightly lower than that of TECs with symmetric electrodes (Figure 5e). Thus, the solidified redox couples, including iron phytate and the alternative‐ferrocene, could have tremendous potential in the optimization of new electrodes in device design and overcoming the mass transport limitations.

Although the thermo‐voltage of the ionic thermoelectric material could be 2–3 orders of magnitude higher than that of the electronic thermoelectric species, the intrinsic non‐redox electrolytes could not provide energy continuously, and the charge was stored as the double layers (Figure 5e). A few references support high thermopower with a low energy density (for example, energy density: 0.006 J m^−2^, thermopower: 10 mV K^−1^, PEO‐NaOH;^[^ ^23^ ^]^ energy density: 0.26 J m^−2^, thermopower: 14.8 mV K^−1^, SiO_2_‐IL;^[^ ^46^ ^]^ energy density: 2 J m^−2^, thermopower: 12.6 mV K^−1^, PSS‐H‐GO^[^ ^47^ ^]^).

Theoretically, the maximum potential difference induced in a thermocell is determined by the redox couple, which undergoes an entropy change due to oxidation or reduction. Hence, the new types of ionic thermoelectric cells demonstrated a high thermopower and improved energy density. The most representative study done by the *Liu* group assembled Gelatin‐KCl‐K_3_Fe(CN)_6_/K_4_Fe(CN)_6_ ionic thermoelectric materials by combining thermodiffusion and thermogalvanic effects, achieving a high thermopower of 17 mV K^−1^ and high energy density of 18.3 J m^−2^ for 2 h.^[^ ^14^ ^]^ The energy density with 3D Au/Cu electrodes improved to 80 J m^−2^ in 2 h,^[^ ^24^ ^]^ and the recent pioneering work was improved to 198 J m^−2^ (2 h) by enhancing the Δ*T*_max_.^[^ ^25^ ^]^ Although the synergistic effect of thermodiffusion and thermogalvanic appears to dominate the thermoelectric conversion process, the resultant entropy change could be still influenced by a variety of factors, including the charge transfer overpotentials caused by the contact of redox couple at the electrode interface and mass transport overpotentials relevant to the migration or structural change of redox species in electrolytes. For this reason, we employed a discontinuous interface modification strategy to load the redox‐pairs onto symmetric and asymmetric electrodes. Both thermocells exhibited effectiveness for low‐grade heat harvesting properties, and an ultra‐high average thermopower of 26.7 mV K^−1^ was achieved in TECs with both symmetric electrodes and with asymmetric electrodes. Specifically, the iron (II/III) phytate‐integrated cell system afforded a record‐high energy density of 2451 J m^−2^ in 2 h (Δ*T* = 15 K) (energy density of 2213 J m^−2^ for TECs with asymmetric electrodes), which was almost one order of magnitude higher than previously reported values. Consequently, the performance of the thermocell can be finely controlled by varying the solidified redox couples, and such a simple layer by layer treatment could be diversely used for future integration or packaging.^[^ ^26^, ^48^ ^]^

### 2.6 Carnot‐Relative Efficiency and Thermal Charge/Electrical Discharge Cycle Stability of TECs with Symmetrical Electrodes

As the demonstration for proof of concept, the most challenging limitation lies in its very low energy conversion efficiency (in comparison with Carnot engine) and the value in most of reported thermocell devices is less than 1%. In this study, the Carnot‐relative efficiency of TECs with symmetrical electrodes was explored. The full consideration of the saturated voltage build‐up time (*t*_i_), power output time (*t*_ii_), and the average output power density (*P*_ave_) into the Carnot‐relative efficiency (*η*_r_) can be integrated into the following function:^[^ ^24^ ^]^

(3) $$\eta_{r}=\frac{\eta }{\eta_{\mathrm{Carnot}}}=\frac{\int_{0}^{t_{\mathrm{ii}}}v\left(t\right)\times I\left(t\right)\mathrm{dt}}{\lambda \frac{\Delta T}{d}\times A\times (t_{i}+t_{\mathrm{ii}})}÷\left(\frac{\Delta T}{T_{H}}\right)\frac{t_{\mathrm{ii}}}{t_{i}+t_{\mathrm{ii}}}\frac{P_{\mathrm{ave}}}{\Delta T^{2}}T_{H}\frac{d}{\lambda }$$

**Figure 6:**
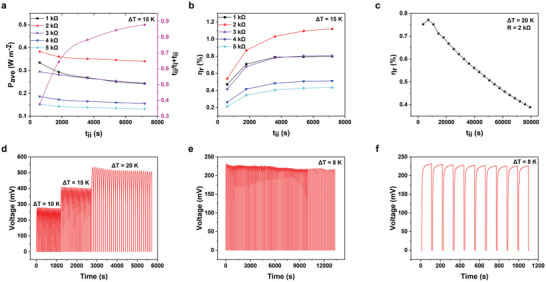
Carnot‐relative efficiency and thermal charge/electrical discharge cycle stability of TECs with symmetrical electrodes. a) The curves of *P*_ave_ and *t*_ii_/*t*_i_ + *t*_ii_ versus *t*_ii_ (*t*_i_ is the saturated voltage build‐up time, *t*_ii_ is the power output time). b) The values of *η*_r_ versus *t*_ii_ of the TECs with symmetrical electrodes under *T*_H_ = 30 °C and *T*_C_ = 15 °C. c) The values of *η*_r_ versus *t*_ii_ of the TECs with symmetrical electrodes under *T*_H_ = 35 °C and *T*_C_ = 15 °C. d) Quasi‐continuous thermal charge/electrical discharge process for the TECs with symmetrical electrodes measured for 30 cycles under variable *T*_H_ (25, 30, and 35 °C) with a fixed *T*_C_ = 15 °C. e) Quasi‐continuous thermal charge/electrical discharge process for the TECs with symmetrical electrodes measured for 120 cycles under *T*_H_ = 23 °C and *T*_C_ = 15 °C. f) The first 10 cycles of the above 120 cycles.

*v*(t) and *I*(t) are the time‐dependent voltage and current during the power output stage. *A*, *d*, *λ*, and *T*_H_ refer to the area, thickness, thermal conductivity, and hot side temperature, respectively. The *λ* value is calculated in Figure S19, Supporting Information. The *t*_ii_/(*t*_i_ + *t*_ii_) is defined as the time factor, in which *t*_i_ is the saturated voltage build‐up time and *t*_ii_ the power output time. *P*_ave_ is related to *t*_ii_, so we set the *t*_ii_ as 600, 1800, 3600, 5400, and 7200 s. The curve of *P*_ave_ and *t*_ii_/(*t*_i_ + *t*_ii_) versus *t*_ii_ are provided in **Figure** **6**a, in which the arithmetic product of two factors including *P*_ave_ and *t*_ii_/(*t*_i_ + *t*_ii_) increases with the extension of power output time *t*_ii_. According to the above experimental data, the *η*_r_, which is positively correlated with *t*_ii_, is calculated in Figure 6b. For the power output to an external resistance of 2 kΩ under *T*_H_ = 30 °C and *T*_C_ = 15 °C, the Carnot‐relative efficiency was 1.12% (*t*_charge_ = 1000 s, *t*_discharge_ = 7200 s). As shown in Figure 6c, the Carnot‐relative efficiency of the TECs with symmetrical electrodes has a parabolic behavior and reaches its maximum value 0.77% at an external resistance of 2 kΩ for 2 h via the temperature gradient of Δ*T* = 20 K (*T*_H_ = 35 °C; *T*_C_ = 15 °C). After 20 h of continuous operation, the Carnot‐relative efficiency can still be maintained to exceed 0.4% at Δ*T* = 20 K.

The thermo‐ionic charging and electronic discharging process as the working principle has also been clarified. The cold side was kept at 15 °C, and the hot side was varied from 25, 30, to 35 °C (Δ*T* = 10, 15, and 20 K). The heating treatment thermally charged the electrode in 1000 s to reach a near‐saturated voltage, and the following quasi‐continuous operation mode was effective. When Δ*T* was equal to 10 K (or 15 and 20 K), the ions with opposite charges near the two electrodes generated a voltage of 281 mV (411 and 524 mV) in 40 s (45 and 90 s) and was then electrically discharged to 0 mV in 5 s (5 s and 10 s) for 30 cycles (Figure 6d). There were no apparent changes for the voltages, and the high stability supported the reversibility of the temperature‐dependent features. The favorable reproducibility of the device function has also been verified in a narrow temperature range (Δ*T* = 8 K; *T*_H_ = 23 °C and *T*_C_ = 15 °C). The accumulated cations/anions at the two sides resulted in thermo‐voltage (232 mV) in a fast mode (100 s), and the voltage decay to zero proceeded in 10 s for 120 cycles (Figure 6e,f). The relatively stable voltage profile suggested TECs with symmetrical electrodes could work well with durability under variable conditions.

### 2.7 Application of TECs with Symmetrical Electrodes and Mechanical Properties of the Ionic Hydrogel

Due to the ultrahigh output power and sustainable thermo‐electrochemical performance, the promising capability of the optimized system in harvesting low‐grade thermal energy has been demonstrated. A prototype module consisting of six units from TECs with symmetrical electrodes was fabricated, and the multiple units were connected in series. The total open‐circuit voltage was increased to 2.3 V at an applied Δ*T* = 15 K (*T*_H_ = 30 °C; *T*_C_ = 15 °C). A LED lamp was directly powered without any DC‐DC voltage boosters (Figures S20 and S21, Supporting Information). Since flexible electronics play important roles in medical engineering, wearable devices, and artificial intelligence, the intrinsic mechanical property of the ionic hydrogel with a size of 25 mm × 10 mm × 2 mm was studied. As illustrated in Figure S22, Supporting Information, the sample was subjected to tensile measurements and its excellent robustness gave rise to a high elongation at break of 1129% with a tensile rate of 100 mm min^−1^. Such a value is much larger than the extension of the human skin. It could also withstand large deformations from twisting or curling treatments (Figures S23 and S24, Supporting Information).

## 3 Conclusion

As the fundamental problem in thermocell energetics and kinetics is predominantly related to the well‐balanced assembly of the electrolyte and electrode, efforts have been concentrated on synergistic optimization for the energy conversion efficiency of single cells. A “one stone kills two birds” strategy was employed here to yield a remarkable voltage of ≈ 0.4 V, an excellent instantaneous output power density (20.26 mW m^−2^ K^−2^), a record‐high 2 h output energy density (2451 J m^−2^) under *T*_H_ = 30 °C and *T*_C_ = 15 °C, and an ultrahigh Carnot‐relative efficiency of 1.12%. Both the primary factors, including temperature‐dependent Carnot‐relative efficiency and independent output power density, support the superiority of the thermocells. The fine control over the ionic hydrogel of the electrolyte and carbon cloth/solidified iron (II/III) phytate composite electrodes has led to a breakthrough not only in surpassing the thermodynamic limit (voltage) but also in facilitating the microscale reaction kinetics of the redox pair onto the electrode. The concentration gradient difference that arises in the electrolyte caused the thermodiffusion effect, and the fast mobility of protons was driven by strong interaction with numerous amide units (versus to a single amide bond) in polyacrylamide gels.

Moreover, the advantage of the traditional concept of asymmetric framework in electrode design has been demonstrated to be negligible within the field of ionic‐type of thermoelectric materials. Herein, the integration of the ferrocene/ferrocenium couple onto the electrode has achieved a variety of favorable parameters, such as instantaneous power density (15.02 mW m^−2^ K^−2^) or an energy density of 2213 J m^−2^ (exposure to an external resistance of 2 kΩ; Δ*T* = 15 K) and verified the reliability of the reversible one‐electron oxidation/reduction. To represent the practical value of such a structural design, six TECs with symmetrical electrodes were in series, and the integrated system generated a high output voltage of 2.3 V. An LED lamp can be lit up for as long as 2 h under the temperature gradient of 15 K (*T*_H_ = 30 °C; *T*_C_ = 15 °C). The feasibility of the module for real implementation could be groundbreaking for the transformation from lab‐scale sample to commercially available products.

## 4 Experimental Section

### Materials

Ferrocene (99%), Acrylamide (99%), *N*, *N*′‐Methylenebisacrylamide (99%), 2‐hydroxy‐2‐methylpropiophenone (97%), and *N*‐methylpyrrolidone (99.5%) were purchased from Shanghai Macklin Biochemical Co., Ltd. Iron (III) chloride and iron (II) chloride were purchased from Guangzhou Chemical Reagent Factory. Phytic acid (70%) was provided by Shandong Yousuo Chemical Technology Co., Ltd. Carbon black was purchased from Guangdong Canrd New Energy Technology Co., Ltd. Polyvinylidene fluoride was provided by Arkema (Shanghai) Distribution Co., Ltd. Carbon cloth was purchased from Toray Industries (China) Co., Ltd.

### Preparation Process of the Ionic Hydrogel

First, 10 g acrylamide, 0.015 g *N, N*′‐Methylenebisacrylamide, and 50 µL 2‐hydroxy‐2‐methylpropiophenone were added to 30 mL deionized water and stirred for 30 min. Then, the solution was poured into a cylindrical quartz mold with a diameter and height of 1.5 cm and irradiated with 365 nm ultraviolet light with a power density of 2 W cm^− 2^ for 4 min to obtain the ionic hydrogel. Finally, the ionic hydrogels were immersed in a phytic acid (0.10 mol L^−1^) solution for 72 h.

### Preparation Process of the Carbon Cloth/Iron (II/III) Phytate Composite Electrode

First, 0.52 g iron (III) chloride and 0.41 g iron (II) chloride were added to 50 mL deionized water, 3 mL phytic acid (70%) was added dropwise while stirring, and the suspension was continually stirred for 30 min. Next, the suspension was transferred to a centrifuge tube and centrifuged at 8000 r min^−1^ for 10 min to obtain iron (II/III) phytate. Then, 0.35 g iron (II/III) phytate, 0.10 g carbon black, and 0.05 g polyvinylidene fluoride were added to 3 mL *N*‐methylpyrrolidone and stirred for 4 h to obtain the slurry. Finally, the slurry is applied evenly to the surface of the carbon cloth and dried at room temperature for 72 h. The carbon cloth had a length of 4 cm and width of 2.5 cm, and a mass loading of iron (II/III) phytate is about 5 mg cm^−2^.

### Preparation Process of the Carbon Cloth/Ferrocene Composite Electrode

First, 0.35 g ferrocene, 0.10 g carbon black, and 0.05 g polyvinylidene fluoride were added to 3 mL *N*‐methylpyrrolidone and stirred for 4 h to obtain the slurry. Then, the slurry was applied evenly to the surface of the carbon cloth and dried at room temperature for 72 h. The carbon cloth had a length of 4 cm and a width of 2.5 cm, and a mass loading of ferrocene is about 5 mg cm^−2^.

### Preparation Process of the TECs with Symmetrical Electrodes

The ionic hydrogel was sandwiched between the carbon cloth/iron (II/III) phytate composite electrodes.

### Preparation Process of the TECs with Asymmetric Electrodes

The ionic hydrogel was sandwiched between the carbon cloth/iron (II/III) phytate composite electrode and the carbon cloth/ferrocene composite electrode.

### Characterization

The XPS full spectra and XPS Fe 2p spectrum of the carbon cloth/iron (II/III) phytate composite electrode were tested by a Thermo ESCALAB 250XI. The thermopower was derived by characterizing the temperature difference and output voltage of the TECs. The temperature difference was attained with a temperature controller. The output voltage was obtained by an electrochemical workstation (CHI760E). The thermal conductivity was measured by a transient plane heat source method (TPS2500S, Sweden). The mechanical property of the ionic hydrogel was tested by an electronic universal testing machine (Byes2005) with a tensile rate of 100 mm min^−1^.

## Conflict of Interest

The authors declare no conflict of interest.

## Supporting information

Supporting Information

## Data Availability

The data that support the findings of this study are available from the corresponding author upon reasonable request.
