# Supplementary material for: Gigantic and Continuous Output Power in Ionic Thermo‐Electrochemical Cells by Using Electrodes with Redox Couples
Source: Adv Sci (Weinh). 2023 Aug 1;10(29):2303407. doi: 10.1002/advs.202303407 (PMC10582453; doi:10.1002/advs.202303407)
Supplement: Supplementary file 1 — Supporting Information [file ADVS-10-2303407-s001.pdf]

ADVANCED SCIENCE
Open Access

Supporting Information

for *Adv. Sci.*, DOI 10.1002/advs.202303407

Gigantic and Continuous Output Power in Ionic Thermo-Electrochemical Cells by Using Electrodes with Redox Couples

Wencong Zhang, Liyu Qiu, Yongjian Lian, Yongqiang Dai, Shi Yin, Chen Wu, Qianming Wang, Wei Zeng* and Xiaoming Tao*

Supporting Information

Gigantic and continuous output power in ionic thermo-electrochemical cells by using electrodes with redox couples

Wencong Zhang, [a,b] *Liyu Qiu,* [a] *Yongjian Lian,* [c] *Yongqiang Dai,* [b] *Shi Yin,* [c] *Chen Wu,* [b] *Qianming Wang,* [a,*] *Wei Zeng,* [b,*] *and Xiaoming Tao* [d]

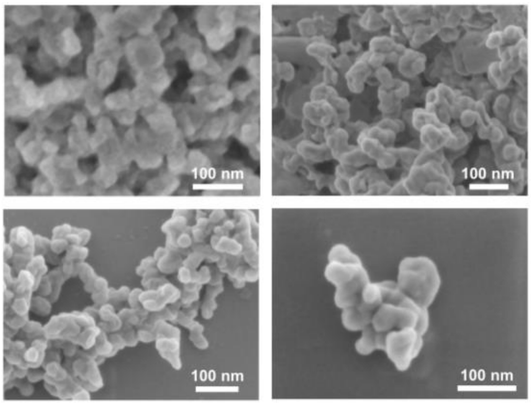

Figure S1. Surface microstructure of the carbon cloth/iron (Ⅱ/Ⅲ) phytate composite electrode.

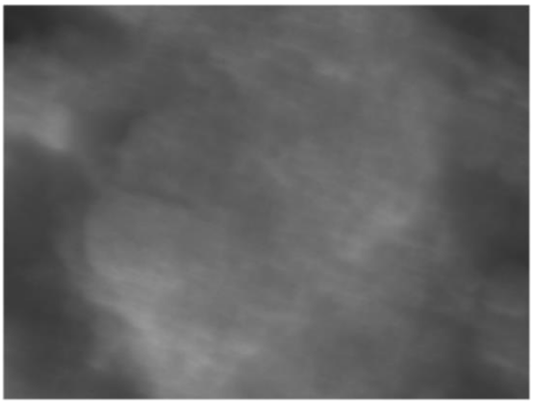

Figure S2. Surface microstructure of the carbon cloth/iron (II/III) phytate composite electrode.

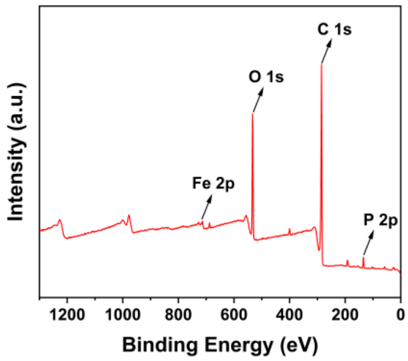

Figure S3. XPS full spectra of the carbon cloth/iron (II/III) phytate composite electrode.

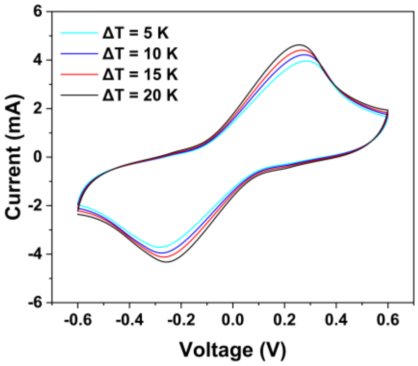

Figure S4. CV curves of the TECs under variable T_H (20 °C, 25 °C, 30°C, and 35 °C) with a fixed T_C = 15 °C.

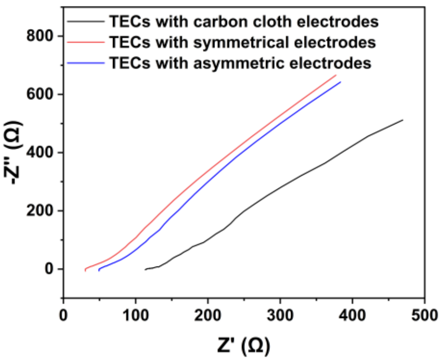

Figure S5. Nyquist plots of the TECs.

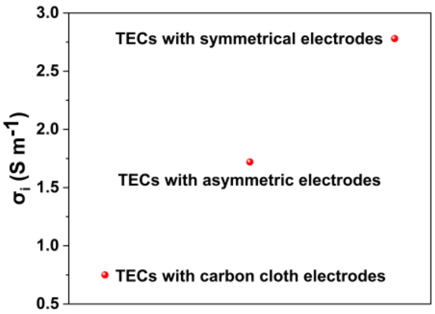

Figure S6. Ionic conductivity of the TECs.

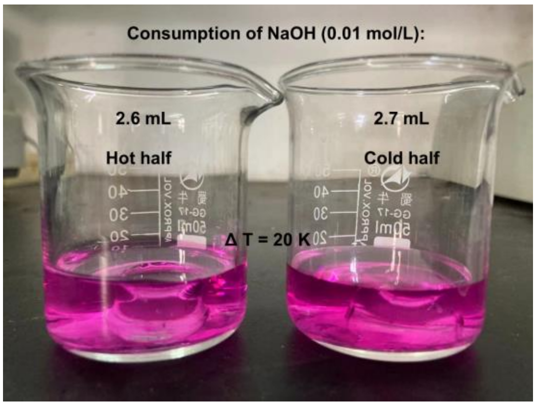

Figure S7. Titration of H^+ concentrations of the hot/cold halves of the hydrogel after thermodiffusion.

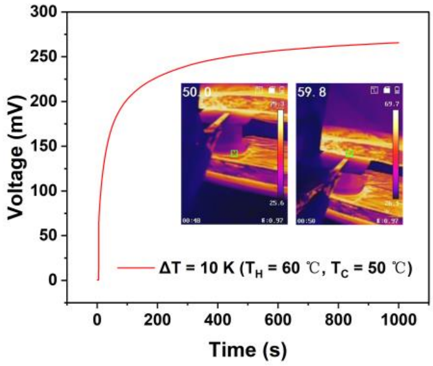

Figure S8. Open-circuit voltage of the TECs under T_H = 60°C and a T_C = 50 °C.

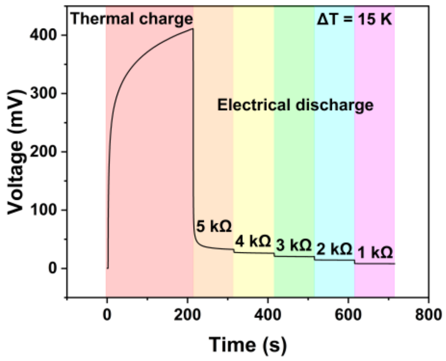

Figure S9. Thermal charge and electrical discharge of the TECs with carbon cloth under T_H = 30 °C and T_C = 15 °C.

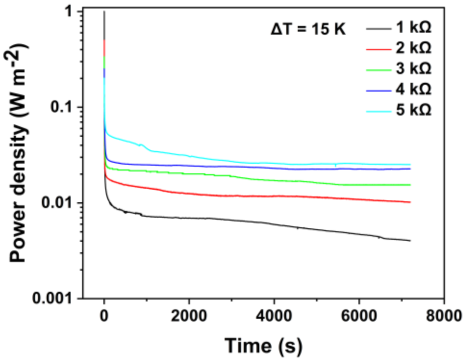

Figure S10. Output power density of the TECs for 2 h at different external resistances with carbon cloth under T_H = 30 °C and T_C = 15 °C.

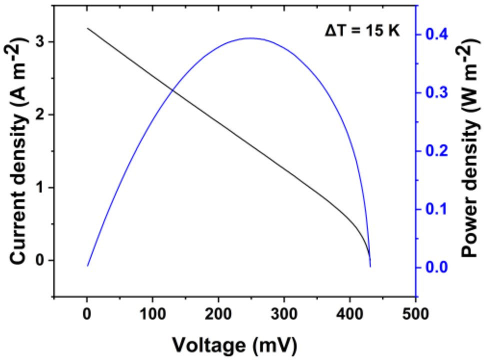

Figure S11. Voltage and output power density versus output current density of the TECs with carbon cloth under T_H = 30 °C and T_C = 15 °C.

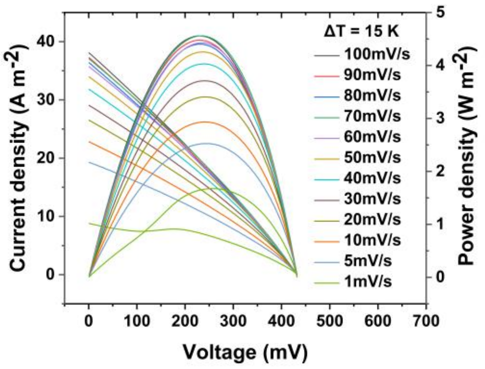

Figure S12. Voltage and output power density versus output current density of the TECs with symmetrical electrodes under T_H = 30 °C and T_C = 15 °C at various sweep speeds.

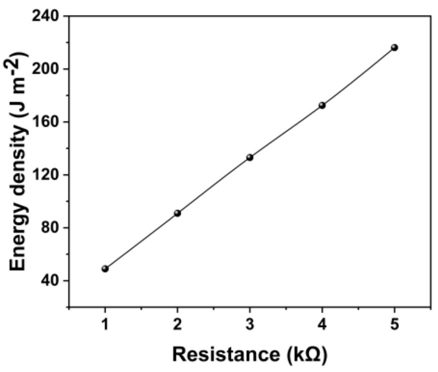

Figure S13. Corresponding energy density of the TECs for 2 h at different external resistances with carbon cloth under T_H = 30 °C and T_C = 15 °C.

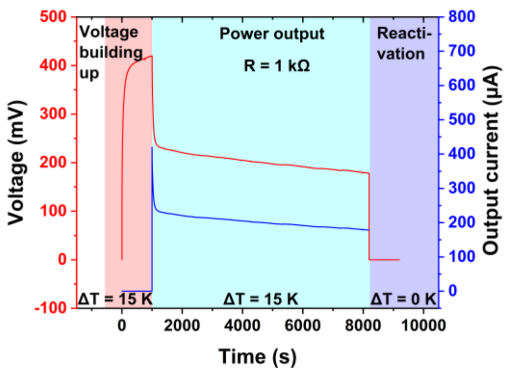

Figure S14. The measured voltage and current curves of the TECs at the external resistance (1 kΩ) with symmetrical electrodes under T_H = 30 °C and T_C = 15 °C.

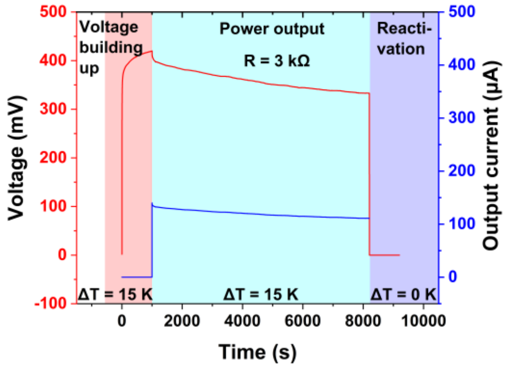

Figure S15. The measured voltage and current curves of the TECs at the external resistance (3 kΩ) with symmetrical electrodes under T_H = 30 °C and T_C = 15 °C.

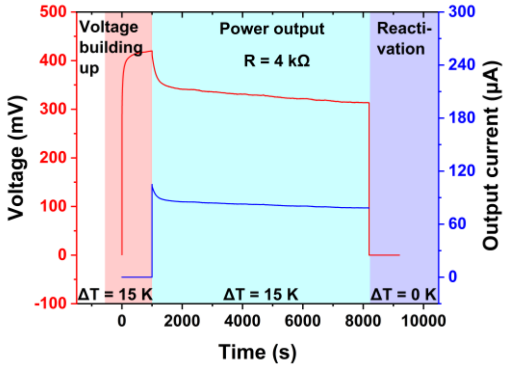

Figure S16. The measured voltage and current curves of the TECs at the external resistance (4 kΩ) with symmetrical electrodes under T_H = 30 °C and T_C = 15 °C.

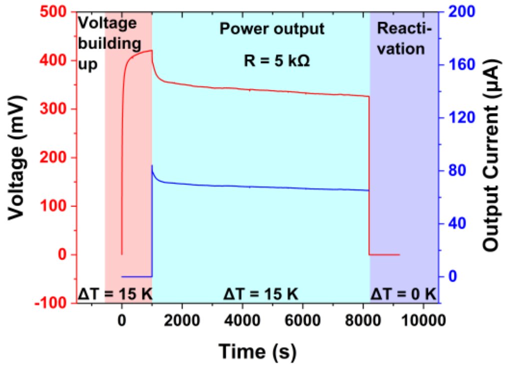

Figure S17. The measured voltage and current curves of the TECs at the external resistance (5 kΩ) with symmetrical electrodes under T_H = 30 °C and T_C = 15 °C.

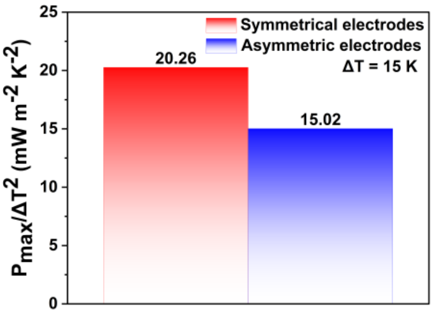

Figure S18. Instantaneous power density ($P_{max}/\Delta T^2$) of the TECs with symmetrical electrodes and the TECs with asymmetric electrodes under T_H = 30 °C and T_C = 15 °C.

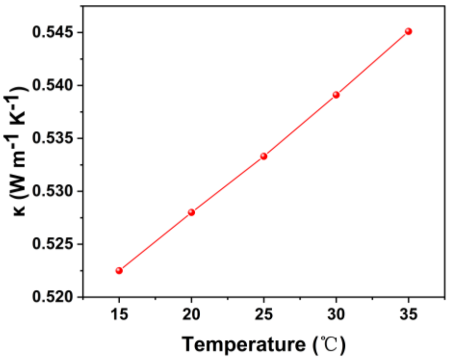

Figure S19. Thermal conductivity of the ionic hydrogel at different temperature (15 °C, 20 °C, 25 °C, 30°C, and 35 °C).

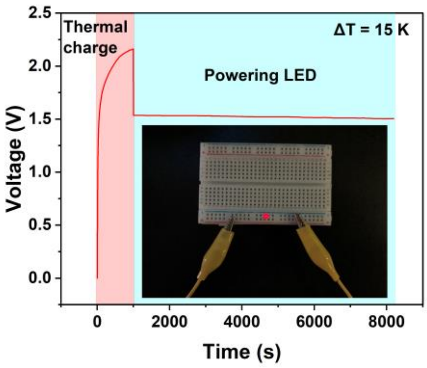

Figure S20. Voltage generated from six TECs with symmetrical electrodes in series under T_H = 30 °C and T_C = 15 °C.

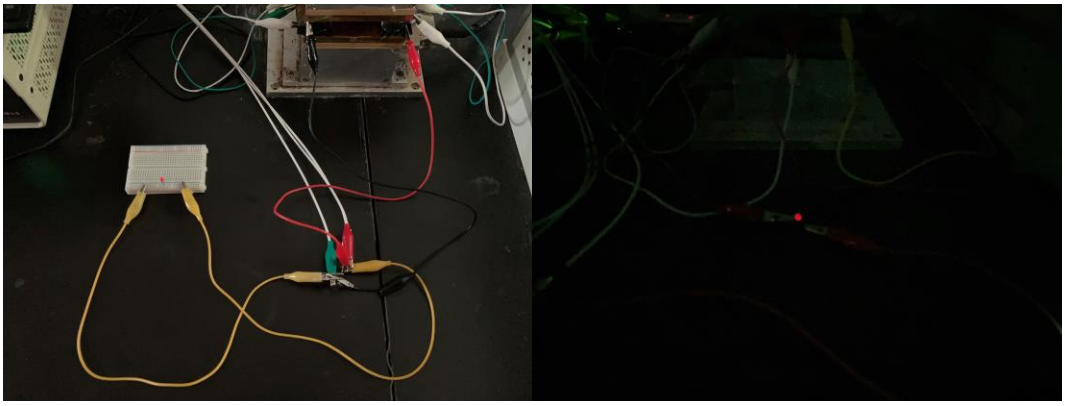

Figure S21. Powering a LED by using six TECs with symmetrical electrodes in series under T_H = 30 °C and T_C = 15 °C.

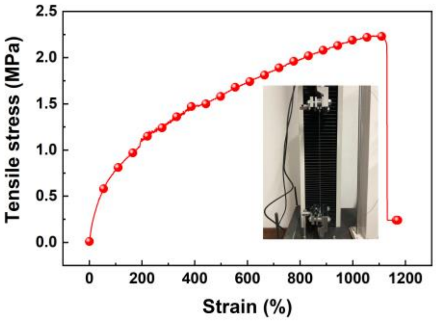

Figure S22. Tensile curve of the ionic hydrogel.

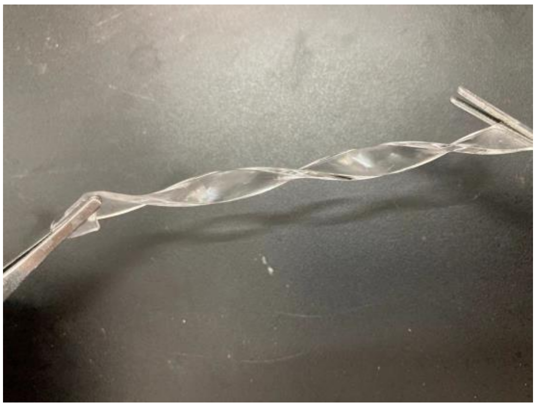

Figure S23. Photograph of the ionic hydrogel under curling treatment.

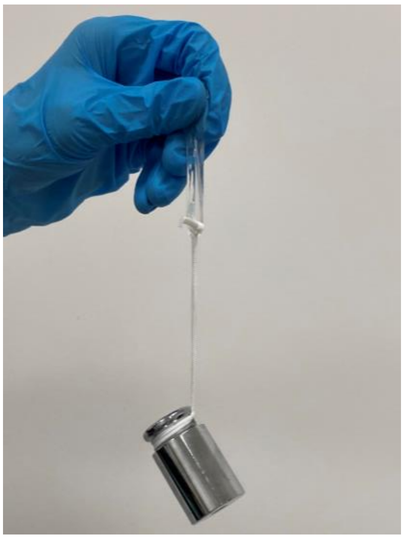

Figure S24. Photograph of the ionic hydrogel withstanding a load of 200 g.

All the following data are calculated using the Gaussian 09 program under M062X/6-31+G*. The extension of repetitive polyacrylamide units (from acrylamide monomer to acrylamide trimer) possesses stronger binding energy of H^+ to $-CONH_2$ (Table S1), indicating that the migration path entangled along with the polyacrylamide chain is reliable.

Initial molecular structure	Final molecular structure	Binding energy
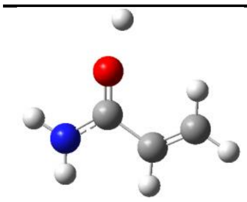	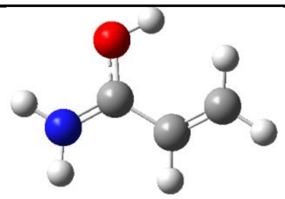	-195.293 kcal/mol
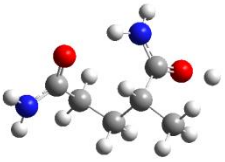	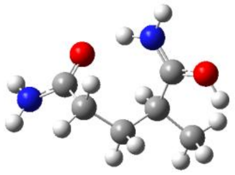	-208.507 kcal/mol
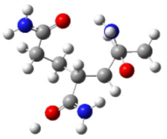	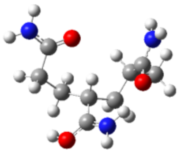	-209.655 kcal/mol

Table S1. Initial molecular structure, final molecular structure, and binding energy of H^+ with acrylamide monomer, acrylamide dimer, and acrylamide trimer calculated by density functional theory (DFT).
